# Soluble CD30 is a new biomarker to predict two-year overall survival of adult T cell leukemia/lymphoma patients

**DOI:** 10.1186/1742-4690-11-S1-P2

**Published:** 2014-01-07

**Authors:** Ratiorn Pornkuna, Shigeki Takemoto, Michihiro Hidaka, Yoshio Haga

**Affiliations:** 1 Department of International Medical Cooperation, Graduate School of Medical Sciences, Kumamoto University, Kumamoto, Japan; 2 Institute for Clinical Research, National Hospital Organization Kumamoto Medical Center, Kumamoto, Japan; 3 Department of Hematology, National Hospital Organization Kumamoto Medical Center, Kumamoto, Japan

## Introduction

Adult T-cell leukemia/lymphoma (ATLL) caused by human retrovirus, HTLV-1, is a neoplasm of mature T-cell. In Japan, more than a thousand patients with ATLL die every year incurable with median survival time less than one year. We aim to investigate clinical value of soluble CD30 (sCD30) levels in sera on prediction of short term mortality and overall survival (OS) in ATLL patients.

## Methods

Between September 2005 and December 2010, a prospective cohort study was done in 60 ATLL patients treated with chemotherapy or allogeneic hematopoietic stem cell transplantation (HSCT) at the Department of Hematology, National Hospital Organization Kumamoto Medical Center, Japan. Univariate analysis of OS was performed using the Kaplan-Meier method to estimate survival probabilities in patient subgroups, and the log-rank test was used for statistical comparisons.

## Results

In the patients undergoing chemotherapy, pre-treatment status of BUN, creatinine, AST, ALT ALP, total bilirubin, LDH, serum albumin, corrected calcium, platelet, percentage of malignant cells in peripheral blood, serum sCD30 level, serum soluble IL-2 receptor level, performance status and Charlson’s comorbidity index were the risk factors of two-year OS. In patients undergoing HSCT, pre-treatment status of source of stem cells, HLA matching, BUN, LDH, corrected calcium, CRP, serum sCD30 level, and Charlson’s comorbidity index were the significant risk factors of two-year OS. Our result suggested that sCD30 was the common significant risk factor of chemotherapy and HSCT in two-year OS.

## Conclusion

sCD30 may be a new biomarker for newly-diagnosed ATLL. The combination of various factors can provide useful information of OS prediction.

